# Emerging Scientists in Analytical Sciences: Zhuoheng Zhou

**DOI:** 10.1002/ansa.202400057

**Published:** 2024-10-29

**Authors:** Zhuoheng Zhou

**Affiliations:** ^1^ Department of Chemical Engineering Vrije Universiteit Brussel (VUB) Brussels Belgium

Through a collection of editorials titled “Emerging Scientists in Analytical Sciences,” we aim to spotlight promising individuals who are actively engaged in the realm of analytical sciences. For this editorial, we invited Zhuoheng Zhou who recently submitted his PhD thesis at the Vrije Universiteit Brussel.

## 1 How Did You Get Involved in the Field of Analytical Sciences?

During my junior high school years, I was very grateful to have attended a scientific event named “*knowing about colours*”, where teachers demonstrated the light dispersion through a prism and the separation of chlorophyll green and carotenoid yellow by a piece of filter paper (until many years later that I realized it was the “*Eureka moment*” of chromatography). I was fascinated by the hidden complexity of mother nature, as much as the fact that it tends to leave us a backdoor to have a glimpse of the core. There was no such thing as attractive to a teenager as solving riddles with obscure hints scattered here and there. I later read about the biography of Antoine Lavoisier and his illumination on the essence of combustion [1]. The profound impact of his quantitative and analytical thinking on modern chemistry research made me realize that analytical science is *de facto* pro‐science, the philosophies, methodologies and techniques it comprises are primers for any scientific research activities. To understand something, you have to “see” or “feel” it first.

I also owe my special thanks to Professor Bo Zhang at Xiamen University for hosting me as a research trainee during the summer of my second bachelor's year. This was the first time I had a chance to step on the floor of a research lab and to have a flavour of modern analytical instruments (mainly chromatographs, electrophoresis apparatus and spectrometers). Despite my “greenness”, I was still assigned to a small project on developing a fast reverse‐phase chromatographic method as the second‐separation dimension in a two‐dimensional liquid chromatography (2D‐LC) set‐up, following a first‐dimension strong cation‐exchange separation, for intact protein profiling in velvet antler (a constituent of traditional Chinese medicines). The trainee program had a very generous deadline which permitted me to start with extensive exploratory scouting runs (a nicer way to say “trial‐and‐error”) and to get myself familiarized with various column chemistries and separation conditions (although in a rather primitive and intuitive way). I remember vividly the goose bumps that I had when I saw my first chromatogram on the screen with five distinctive peaks, only five minutes after I manually injected some sort of colourless, transparent, and homogeneous liquid that a senior prepared for me. The joy of knowing the supposedly unknown (more like finishing the last chapter of a detective fiction book) sparked my deep interest in the world of analytical science and particularly separation science up to this day.

## 2 What was the Topic of Your PhD Study?

My PhD thesis comprised of two major parts. The first part revolves around the instrumentation design and optimization for ultra‐high‐pressure LC (UHPLC). We demonstrated the influence of fluidic configurations on the dispersive and thermodynamic properties of chromatography eluents under extremely high‐pressure conditions (1500 bar) [2]. To shed light on the trade‐off between gain in overall efficiency and loss in column‐accessible pressure when narrower tubing and smaller detector cells are used, we have borrowed the principle of the kinetic plot method—which has been mainly applied for column characterization—and extended its application to evaluate the kinetic performances of entire UHPLC system. Based on the insights we gained throughout this project, we have in collaboration with instrument vendors and government labs, successfully established an optimum UHPLC system configuration and applied it for ultra‐high resolution profiling of multi‐class residuals analysis in dairy products [3].

The second part focuses on advancing column technology for low‐flow LC (LF‐LC), as an indispensable chromatography technique coupled to mass spectrometry (MS) for bio‐separations with significantly improved sensitivity. We have developed a protocol [4] describing the fabrication process of monolithic polymer stationary phases in capillary column formats, alongside guidelines on tuning the macropore structure targeting high kinetic performance. The versatility of the developed columns was demonstrated for high‐throughput and high‐resolution LF‐LC bio‐separations of intact proteins, peptides, and oligonucleotides, with verified robustness and repeatability. To further strengthen our understanding of the transport phenomena in polymer monoliths, tomographic reconstruction and stereological analysis were leveraged, to correlate the column bed morphology and kinetic performances for future column designing [5].

## 3 What was Your Biggest Achievement During This Time?

During my PhD, I was fortunate to have time to tidy up some of my research results and turn them into seven peer‐reviewed publications and four international conference presentations. I was also privileged to be interviewed by LCGC as Rising Star of Separation Science in 2023, which offered me a great opportunity to share my work with the community. Besides external recognition, my proudest moments are actually dispersed in the daily research. In the project of a quantitative assessment of the 3D monolithic porous network, we went to great lengths to collaborate with chemical engineers, image analysts, and data scientists to find the proper morphological descriptors that can be used to chromatographically size a monolith, which has long been deemed challenging. We were among the first to explore the possibility of quantifying the Giddings’ trans‐column velocity bias in a monolith, which surprisingly did not differ significantly from that of particulate columns. We believe these novel insights will be the cornerstones for future monolithic column design.

## 4 Who Were the Most Influential People in Your Path as a Scientist?

Besides Professor Bo Zhang who I mentioned supervised my Bachelor final‐year project, I owe my deepest gratitude to my two PhD promotors. Professor Sebastiaan Eeltink, as my main promotor, his dedicated commitment to chromatography, profound knowledge of polymer chemistry, unbelievably hard‐working ethics, detail‐oriented philosophy and admirable people skills have shed tremendous light on every step of my path as a scientist. As a PhD promotor, his generosity truly gives meaning to this title. He would seize every opportunity to introduce myself and my research works to the peer scientists, not only to promote but also to attract potential interests of collaborators and eventually ended up with many joyful and fruitful scientific adventures.

My heartfelt thanks extend to my co‐promotor Professor Gert Desmet, the chromatography “guru”, the living textbook, and my go‐to‐dictionary only one floor away. Whether it was the explanation of various solvent‐strength models on the first day we met or our recent discussion on Giddings’ geometric constants, each scientific exchange with him has been challenging to fully grasp. Yet, the aftermath that lingered in the days following has deepened my understanding of concepts and reshaped my perspective on models.

## 5 Which Technologies are You Currently Using in Your Laboratory?

**FIGURE 1:**
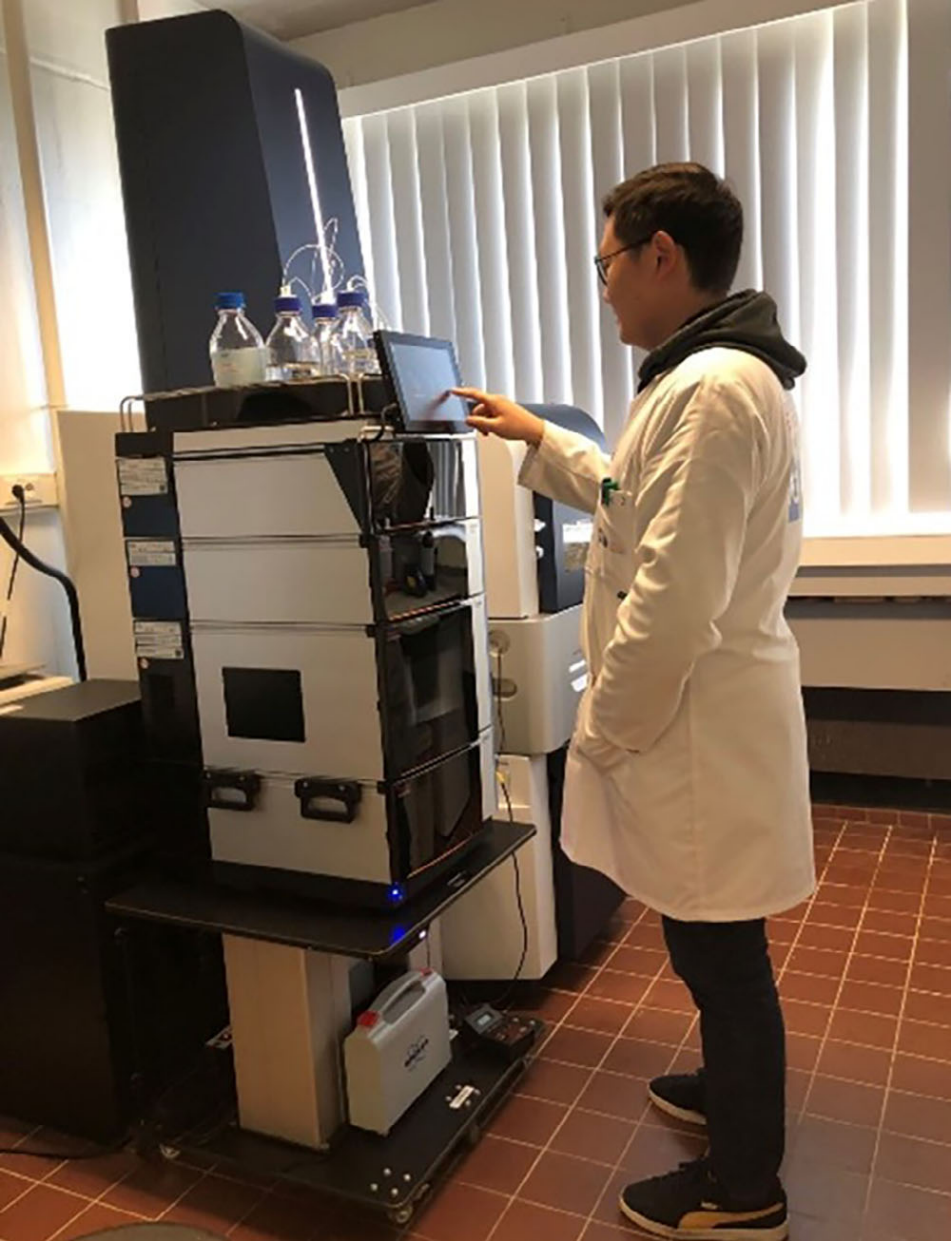
Zhuoheng working with the nanoLC‐timsTOF proteomics platform in the laboratory at Vrije Universiteit Brussel, October 2024.

Currently, we have been exploring the potential of ion‐mobility spectrometry (IMS) in combination with high‐resolution chromatography and MS to further push forward the data quality and analytical throughput of proteomic profiling (Figure 1, also see our recently published review article [6]). IMS, as a gaseous phase separation technique, adds an additional resolving capability after chromatographic elution. This second separation dimension not only isolates the peptide features from matrixial contaminants and differentiates isobaric pairs subject to co‐fragmentation, but also offers the structural elucidation capability to provide additional attributes to increase identification confidence. Yet, similar to two‐dimensional chromatography, the addition of IMS between LC and MS also requires method optimization to prevent separation resolution loss, dilution, and under‐ or over‐sampling.

## 6 Which Current Trends in Analytical Instrumentation are You Interested in?

One clear trend I see in current analytical instrumentation is high throughput, which is especially relevant in industrial chemical analysis where time and operational costs are the main constraints. High‐throughput analysis not only emphasizes the fundamental transformation in analytical principles (e.g., UHPLC in chromatography, data‐independent acquisition in MS) but also requires novel sampling strategies (e.g., compatibility between instrumental analysis with large assays and chemical libraries) and additional technical advancement on minimizing overhead times. What also intrigues me is the blasting data generation permitted by high‐throughput analysis, which requires new perspectives on results interpretation, from traditionally “one‐spectrum‐at‐a‐time” to statistical mining with the assistance of newly advanced machine learning toolboxes.

Another topic I am particularly interested in is the everlasting pursuit of instrument hyphenation to provide complementary characterizations with multiple data dimensions. Analytical science has significantly benefited from the assorts of hyphenated instruments as trivial as meeting an ad‐hoc measuring task and as significant as unlocking a transformative research field (e.g., LC‐MS to proteomics, cytometry by time of flight to single‐cell analysis). These combinations have significantly improved the separation, detection, and analysis of complex samples, making it possible to explore previously uncharted areas in biology, environmental science, and materials research. As these technologies continue to evolve, they are likely to become more integrated, automated, and accessible, contributing to the advancement of scientific discovery and innovation across diverse industries. However, challenges remain in terms of instrument compatibility, cost, and complexity, requiring ongoing research and innovation.

## 7 Where Do You See Yourself in 10 Years?

As a separation scientist, my research interests are rooted deeply in bridging fundamental and technological advancement with real‐world separation challenges. One of my areas of interest is the emerging field of biopharmaceutical analysis. The newly introduced biomolecule‐based drug modalities, for example, recombinant and fusion proteins, monoclonal antibodies (and their drug conjugates), oligonucleotides and mRNA vaccines, come often with high structural complexity, chemical heterogeneity, and low diffusivity, which challenges the conventional separation techniques regarding both selectivity and efficiency. In order to meet the analytical needs of industrial applications, novel separation techniques together with instrumentation engineering are both required to further improve R&D productivity. Conducting research in this field is where I see myself in the next ten years and probably many years to come.

## 8 Can You Say Something About Your Hobbies Outside the Laboratory?

**FIGURE 2:**
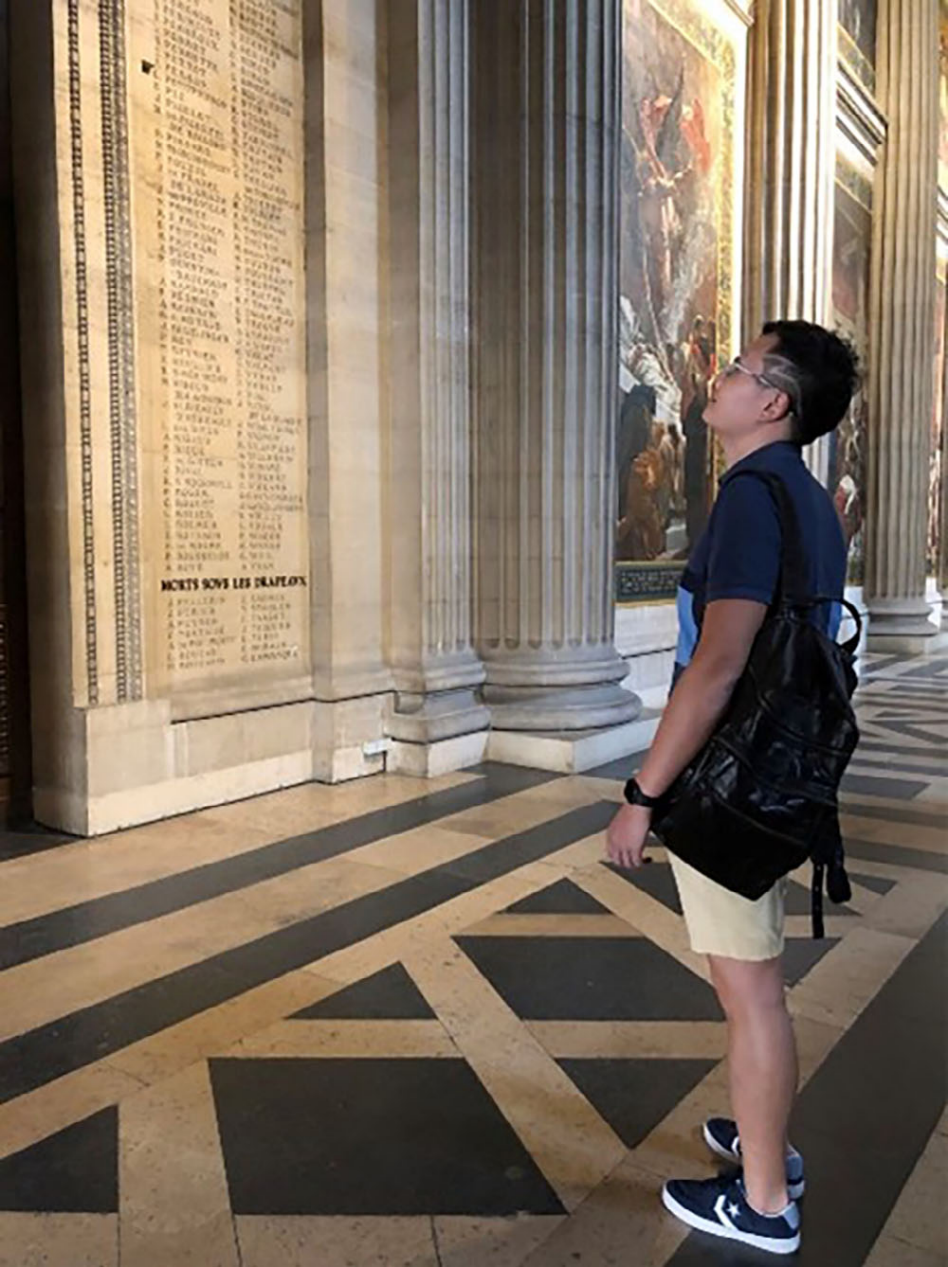
Zhuoheng trying to locate the frescos painted by Jules‐Eugène Lenepveu at The Panthéon Paris, June 2019.

When I am not in the lab, I often see myself in museums or on historical sites, either trying to decipher a Latin inscription or struggling to date an unknown painting based on the brushwork and pigments. Solving puzzles that have been architected by human civilization is as much fun as that orchestrated by nature (Figure 2).

## References

[1] A. Donovan and A. Lavoisier , Science, Administration, and Revolution (Cambridge University Press, 1996).

[2] Z. Zhou , M. De Pra , F. Steiner , G. Desmet , and S. Eeltink , “Assessing Effects of Ultra‐High‐Pressure Liquid Chromatography Instrument Configuration on Dispersion, System Pressure, and Retention,” Journal of Chromatography A 1634 (2020): 461660. 33189961 10.1016/j.chroma.2020.461660

[3] D. Meston , T. Themelis , Z. Zhou , et al., “Development of a Generic Ultra‐High‐Pressure Gradient Liquid‐Chromatography Method Development Protocol: The Analysis of Residual Multi‐Class Antibiotics in Food Products as a Case Study,” Journal of Chromatography A 1684 (2022): 463565. 36274530 10.1016/j.chroma.2022.463565

[4] Z. Zhou , E. F. Hilder , and S. Eeltink , “A Protocol for Fabrication of Polymer Monolithic Capillary Columns and Tuning the Morphology Targeting High‐Resolution Bioanalysis in Gradient‐Elution Liquid Chromatography,” Journal of Separation Science 46 (2023): 2300439. 10.1002/jssc.202300439 37515368

[5] Z. Zhou , T. Themelis , T. Lu , et al., “Quantitative Assessment of the 3D Pore Space and Microglobule Clustering Network to Understand Chromatographic Transport Phenomena in Polymeric Monolithic Columns,” Chemical Engineering Journal 499 (2024): 156200.

[6] S. Perchepied , Z. Zhou , G. Mitulović , and S. Eeltink , “Exploiting Ion‐Mobility Mass Spectrometry for Unraveling Proteome Complexity,” Journal of Separation Science 46 (2023): 2300512. 10.1002/jssc.202300512 37746674

